# How Anterior Cruciate Ligament Injury was averted during Knee Collapse in a NBA Point Guard

**Published:** 2017-03-03

**Authors:** Nathan D Schilaty, Nathaniel A Bates, Aaron J Krych, Timothy E Hewett

**Affiliations:** 1 Department of Orthopedic Surgery, Mayo Clinic, Rochester, Minnesota, USA; 2 Sports Medicine Center, Mayo Clinic, Rochester, Minnesota, USA; 3 Department of Physical Medicine & Rehabilitation, Mayo Clinic, Rochester, Minnesota, USA; 4 Department of Physiology & Biomedical Engineering, Mayo Clinic, Rochester, Minnesota, USA; 5 Department of Biomedical Engineering, The Ohio State University, Columbus, Ohio, USA

## Abstract

Non-contact anterior cruciate ligament (ACL) injuries occur with rapid decelerations and pivoting. A recent injury to a high-level National Basketball Association (NBA) player demonstrated neuromuscular control and injury-sparing mechanisms that resulted in only minor ligament injury to the medial collateral ligament. We analyzed biomechanical mechanisms via publically available orthogonal 2-D video to demonstrate how this potential ACL injury was averted. Analysis of the knee injury mechanism demonstrated that the NBA player experienced low ground reaction force, high sagittal plane flexion, and maintenance of frontal plane stability with neuromuscular control. The outcome of these factors inhibited dynamic valgus collapse of the knee throughout the fall, avoiding ACL injury – a potentially career-altering injury. Many athletes, professional and recreational, will be subjected to similar mechanisms of injury and will have improved outcomes if they can successfully utilize preventive strategies of neuromuscular control to limit injury mechanisms.

## Background

Non-contact ACL injuries are common in competitive-level sports, such as basketball, with movements of rapid decelerations and pivoting. Many athletes are subjected to injurious mechanisms and forces that could potentially cause ACL injury. Proper neuromuscular control can potentially avert ACL injury occurrence. This case study demonstrates:

- The neuromuscular control and biomechanical factors that averted a potentially career-altering ACL injury for a high-level athlete.
- That a dynamic valgus collapse of the knee is a vital characteristic in ACL injury.

## Introduction

During an exceptional season in which a starting National Basketball Association (NBA) point guard demonstrated remarkable skill on the court (averaging 30.1 points per game, 6.7 assists per game, and 5.4 rebounds per game), won the league MVP title, and assisted his team to the 2016 NBA Playoffs, the said player experienced a potentially tragic injury at the close of the 2^nd^ quarter during Game 4 of the opening round series. Moments prior to the injury, an opposing player, rushing down the court, tripped and slid across the floor, leaving a wet residue of sweat on the playing surface. This residue was directly in the path of the starting point guard who was moving at a fast pace down the court in an attempt to take a defensive position and prevent an opposing score in the remaining seconds of the half. The player the point guard was defending pulled up to shoot, leading the point guard to plant his left leg on the slick surface in an attempted rapid deceleration. Immediately, the point guard lost traction causing his left foot to slide out distally from his body. The resultant attempt to hinder the fall with his right leg caused him to incur a right knee injury as he collapsed to the court.

With any high-force knee injury (especially those of rapid deceleration, twisting, and planting as observed frequently in basketball), an anterior cruciate ligament (ACL) or medial collateral ligament (MCL) injury is a reasonable diagnosis to assess. Partial MCL tears (Grade II) are painful, but heal well in the extra-articular environment as clotted blood forms a fibrous network for mending the ligamentous tissue [1]. Even a complete MCL tear (Grade III) can be surgically repaired due to its robust healing potential secondary to the ability of the fibrous clot to form post-surgery [2]. In contrast, the ACL is housed inside of the synovial cavity of the knee, which prevents the establishment of a blood clot due to the regular turnover of the synovium and leaves the ACL without a fibrous network with which to commence healing [3]. Typically, therapies for ACL-injured athletes playing at competitive levels of sport require ACL reconstruction to restore biomechanical integrity of the knee. This reconstruction is invasive and requires approximately 6 to 12 months of intensive rehabilitation [4], following the surgery, but persistent limb asymmetries can be observed even two years after the incident [5,6] Even after successful ACL reconstruction, it is reported that over 50% of young athletes do not return to the same level of play [7]. Furthermore, even following successful ACL reconstruction, early onset osteoarthritis of the knee is prevalent within as great as 90% of patients within 10–15 years leading to decreased quality of life and performance [8–10]. This type of injury would certainly be a hindrance both to the career of a young, starting NBA point guard and to the probability of his team executing a successful playoff run.

However, by a series of fortunate injury-sparing mechanisms for this potentially ACL-injurious incident, the point guard in question only demonstrated a Grade I MCL sprain diagnosed via MRI. Grade I means that the ligament was stretched, but not torn. The team stated that he would be re-evaluated two weeks post injury. During this re-evaluation, the team physicians would determine whether the player was capable of returning to play without likelihood of causing further harm or injury to his knee. The point guard was sidelined for a total of 15 days and missed four playoff games prior to receiving permission from team physicians to return to the court. Although he could not contribute to his team’s playoff run during a brief two week period, both team and player were assured he was not undertaking the intensive physical therapy and surgical reconstruction of his right knee. Such aggressive treatment would equate to the definitive sidelining of the point guard until at least the mid-point of the 2016–2017 basketball season.

## Methods

**Figure 1:**
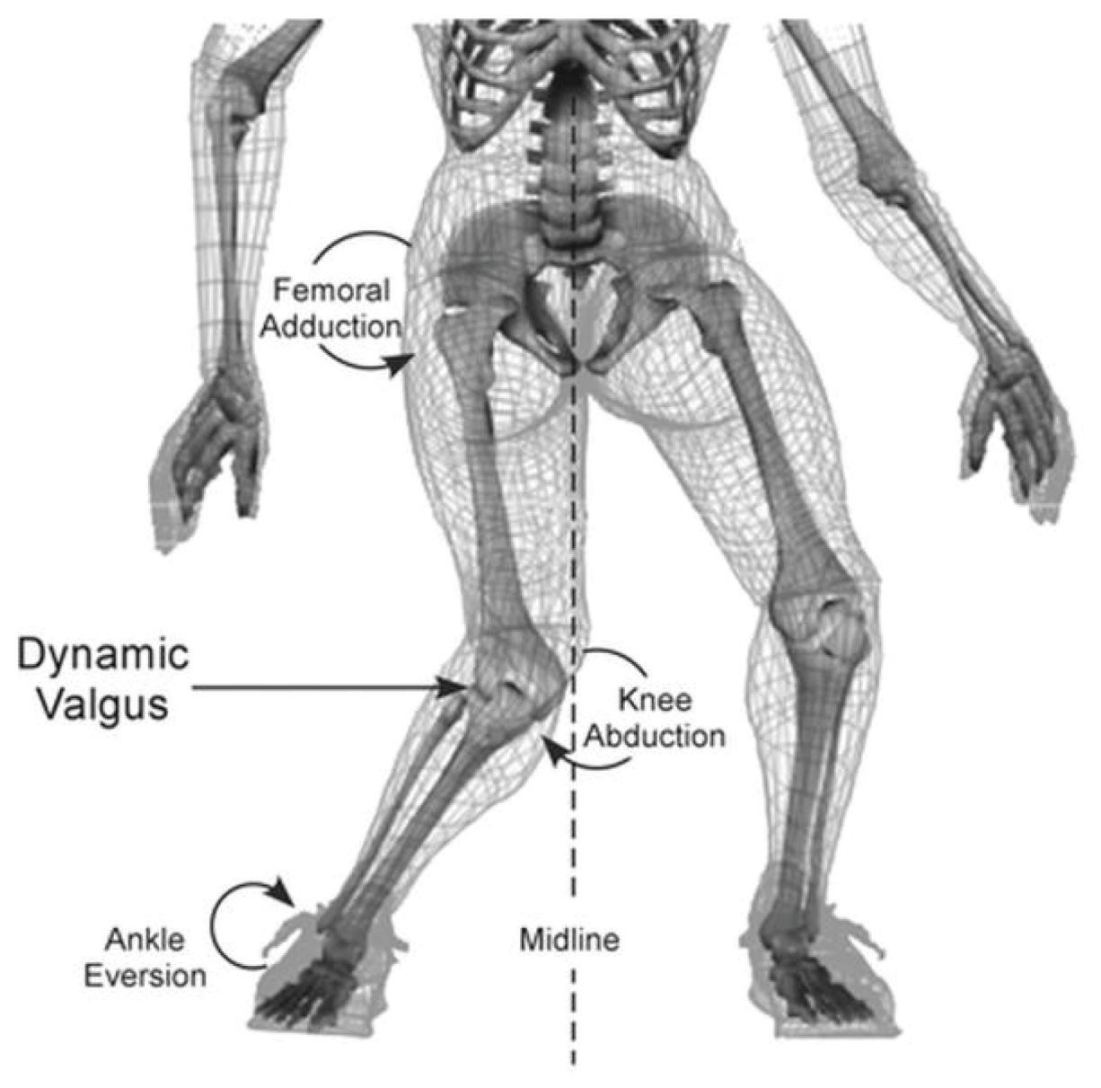
Common mechanism of ACL injury Figure reproduced from Hewett, TE, et al. Biomechanical measures of neuromuscular control and valgus loading of the knee predict anterior cruciate ligament injury risk in female athletes: A prospective study. Am J Sports Med. 2005;33(4):492–501. Used with permission, Sage Publications.

**Figure 2:**
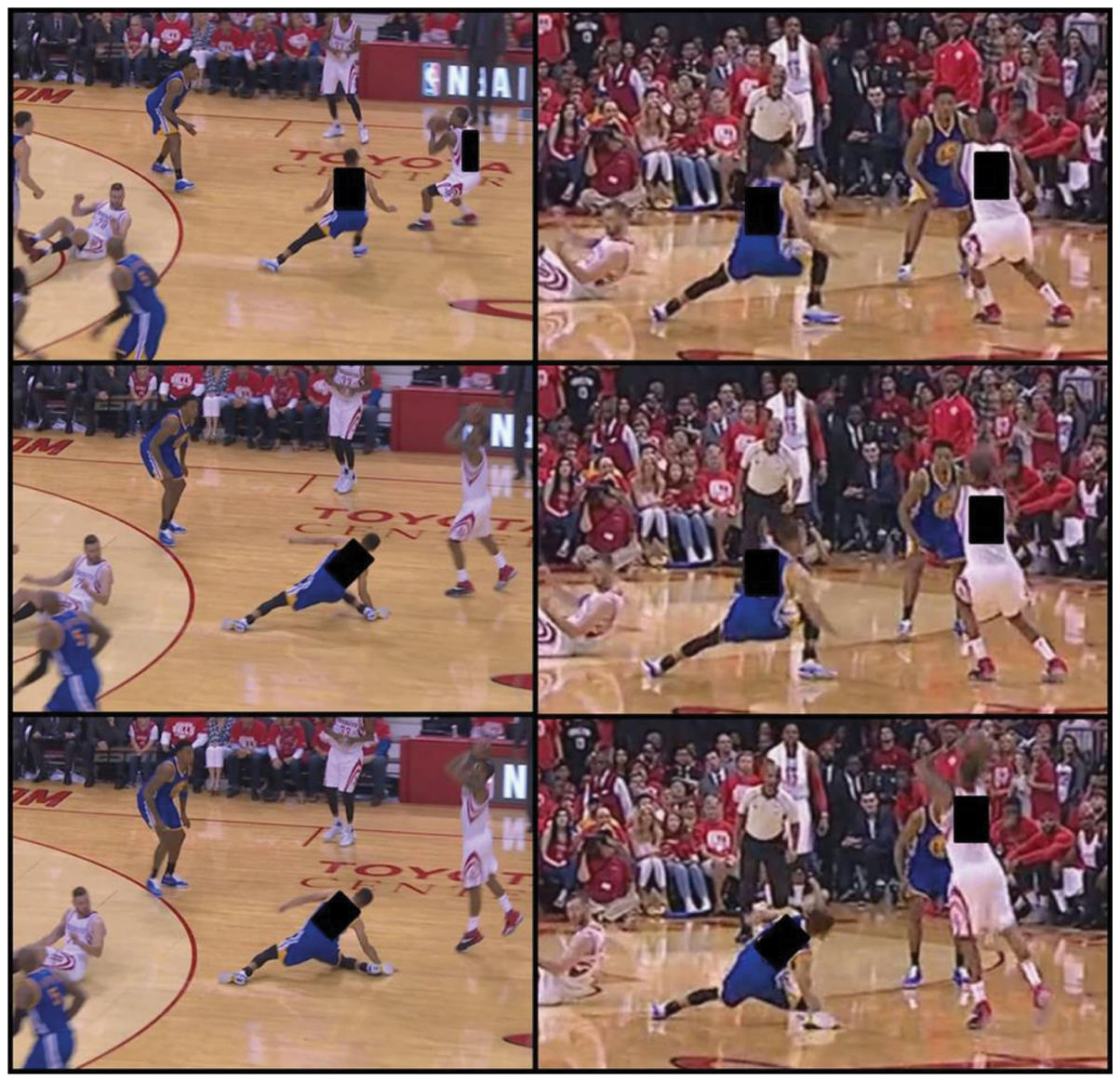
Posterior and lateral views of case report knee injury Successive frames are from top to bottom. Used by permission, NBA.

From both a biomechanical and neuro-mechanical viewpoint, 2-D video can be analyzed for determining mechanism of injury(11) as well as the factors that spared the starting point guard from an ACL rupture (Figure 1) [12]. The 2-D video utilized for analysis in this case study was obtained from publically available video from ESPN. Two orthogonal views of the injury were available for more accurate kinematic analysis. Kinematic assessment was performed on the included images (Figure 2) and performed on Image J 1.50i (National Institutes of Health, Bethesda, MD). Measures not possible with the available images were performed by observation.

## Results

The most prevalent mechanisms of ACL injury entail 1) high ground reaction force; 2) valgus collapse; 3) planted foot (often flat-footed); and 4) the knee near full extension [12,13]. An analysis of the orthogonal 2-D video of the present (Figure 2) injury mechanism indicates that 1) the player slipped on the left foot causing the body weight to shift to the right leg; 2) the right leg was already flexed at nearly 90 degrees, which lowered the respective amount of force that was propagated through the ACL (as the ACL is primarily loaded between 0–30° of knee flexion and is generally unloaded above 45° of knee flexion) [14]; 3) he experienced low ground reaction force due to slippage of the left foot on the wet court, while the right leg was still positioned laterally away from the body in an orientation that prevented it from successfully absorbing all the body’s weight; 4) both legs continued to bear force distribution as the player fell; 5) his neuromuscular system maintained frontal plane stability of the knee, which caused internal rotation at the hip, but inhibited dynamic valgus collapse of the knee throughout the fall. Kinematic assessment demonstrated an initial sagittal plane knee flexion of 105.7° that ended at 115.3° and frontal plane knee valgus of 6.2° that progressed to 13.1°. Transverse plane hip and knee angles were not possible with an absence of an overhead view, but observation demonstrates a large internal hip rotation and minimal knee internal rotation.

## Discussion

The biomechanical construct of the knee allows for ab-/adduction in the coronal plane, flexion/extension in the sagittal plane, and internal/external rotation in the transverse plane. The knee has both extra- and intracapsular ligamentous structures to limit undesirable motion that could lead to injury. The MCL passively limits knee abduction and the lateral collateral ligament passively limits knee adduction. Further passive restraints of knee motion include the ACL, the posterior cruciate ligament (limiting posterior translation of the tibia in relation to the femur) and the knee capsule. Beyond ligamentous stability, the screw-home mechanism of the knee provides mechanical restraint to knee mobility. The screw-home mechanism is provided by a combination of asymmetric femoral condyles and muscular activation, effectively limiting the internal/external rotation of the knee at or near full extension [15,16]. The result of the screw-home mechanism is increased stability during contact with the ground as internal/external rotation is limited. These multiple passive structures and mechanism exist to limit undesired motion at the knee joint, especially during weight-bearing.

As an intracapsular ligament in the femoral notch, the ACL originates on the medial side of the lateral femoral condyle and attaches near the intercondyloid eminence of the tibia, often blending with the anterior horn of the lateral meniscus. With these points of attachment, the ACL passively limits not only the anterior translation of the tibia in relation to the femur, but can also limit knee abduction and internal rotation [17]. These same three planar motions all preload the ligament for injury [18,19], although they preload the ligament in varying degrees. Increased knee abduction leads to an increase in knee abduction moment, which has demonstrated high sensitivity and specificity for ACL injury risk [13]. Interestingly, both *in sim* and *in vitro* testing has demonstrated that knee abduction significantly loads the ACL to strain levels that are not sufficient to compromise the MCL [19,20]. In addition, with anterior tibial translation and internal rotation, the ACL strain reaches levels higher than the MCL [20]. Furthermore, combined torsional loading has a greater biomechanical influence than single plane motion [21,22]. In regard to knee flexion, sagittal plane loading in isolation cannot cause ACL injury [23], and the ACL is unloaded above 50° of flexion [14,24]. With sagittal plan loading unable to cause ACL injury, multiple biomechanical reports have determined that dynamic valgus collapse (combined or coupled motion) of the knee is a major contributor to ACL rupture as this motion rapidly loads the ACL and increases knee abduction moment (Figure 1) [12,19,25,26].

Beyond passive restraints of the knee, the neuromuscular system is vital to providing both reflexive and feedforward signaling to the active muscle restraints of joint stabilization. The contractile and elastic musculature is the preferred tissue to inhibit injurious forces to the passive ligamentous structures and joint capsule. The major musculature of the knee (quadriceps and hamstrings) allow for flexion and extension of the joint. Whereas the hamstrings are agonists to the ACL and will spare anterior tibial translation, the quadriceps are antagonistic and will increase anterior tibial translation and further extend the knee [13]. In addition to providing knee flexion, the medial and lateral insertions of both the hamstrings and gastrocnemii allow for stabilization of knee ab-/adduction and internal/external rotation. If the active muscular restraints can maintain normal alignment of the joint structures during motion, a dynamic valgus collapse (coupled knee abduction, internal rotation, and anterior tibial translation) of the knee can be avoided, sparing the ACL from a traumatic rupture [22]. Multiple reports have demonstrated that preventive interventions employing biofeedback, clinician feedback, and neuromuscular training can successfully reduce the incidence of ACL injury by targeting the above mentioned mechanisms [27–32].

Given the potential injurious situation the NBA point guard in question was subjected to, it is apparent that beyond the unmodifiable factors (i.e. slipping on the floor), he employed protective mechanisms to prevent rupture of the ACL. These included 1) distribution of forces across both legs; 2) high flexion of the knee with likely inhibition of quadriceps contraction; and 3) neuromuscular control to maintain ab-/adduction stability and internal/external rotation of the right knee which allowed for internal rotation to occur at the hip. The kinematic angles measured demonstrated an absence of dynamic valgus knee collapse with associated sparing knee flexion angle. These neuromuscular responses reduced knee abduction, anterior tibial translation, and internal tibial rotation, significantly reducing his risk for sustaining an ACL injury.

## Learning Points

- Although the forces generated and biomechanical mechanisms created a susceptible opportunity for an ACL injury in this NBA point guard, both unmodifiable (e.g. slippery floor) and modifiable factors (potential activation of hamstring musculature to inhibit dynamic valgus collapse of the knee) contributed to only a minor Grade I sprain of the MCL.
- The relatively minor MCL injury spared this extremely high-value point guard from a disastrous end to his otherwise highly successful basketball season. Many athletes, professional and recreational, will be subjected to similar mechanisms of injury and will have improved outcomes if they can successfully utilize neuromuscular control to limit the injury mechanisms.

This “close call” of an averted ACL injury reinforces the need for early preventive neuromuscular training strategies [27,29,32], to continue to limit the incidence of ACL injuries.
